# Comprehensive geological dataset describing a crystalline rock mass for hydraulic stimulation experiments

**DOI:** 10.1038/sdata.2018.269

**Published:** 2018-11-27

**Authors:** Hannes Krietsch, Joseph Doetsch, Nathan Dutler, Mohammadreza Jalali, Valentin Gischig, Simon Loew, Florian Amann

**Affiliations:** 1 ETH Zurich, Department of Earth Sciences, Sonneggstrasse 5, 8092 Zurich, Switzerland; 2 Centre for Hydrogeology and Geothermics, University of Neuchâtel, Rue Emile-Argand 11, CH-2000 Neuchâtel, Switzerland; 3 RWTH Aachen, Engineering Geology and Environmental Management, Lochnerstrasse 4-20, 52064 Aachen,Germany

**Keywords:** Structural geology, Geothermal energy

## Abstract

High-resolution 3D geological models are crucial for underground development projects and corresponding numerical simulations with applications in e.g., tunneling, hydrocarbon exploration, geothermal exploitation and mining. Most geological models are based on sparse geological data sampled pointwise or along lines (e.g., boreholes), leading to oversimplified model geometries. In the framework of a hydraulic stimulation experiment in crystalline rock at the Grimsel Test Site, we collected geological data in 15 boreholes using a variety of methods to characterize a decameter-scale rock volume. The experiment aims to identify and understand relevant thermo-hydro-mechanical-seismic coupled rock mass responses during high-pressure fluid injections. Prior to fluid injections, we characterized the rock mass using geological, hydraulic and geophysical prospecting. The combination of methods allowed for compilation of a deterministic 3D geological analog that includes five shear zones, fracture density information and fracture locations. The model may serve as a decameter-scale analog of crystalline basement rocks, which are often targeted for enhanced geothermal systems. In this contribution, we summarize the geological data and the resulting geological interpretation.

## Background & Summary

Three-dimensional geological models are of foremost importance for many geo-mechanical investigations (e.g., tunneling, hydropower infrastructure, geothermal energy, groundwater), related numerical simulations (e.g., modeling of hydraulic stimulations), and interpretation of complex 3D monitoring data. Since geological information is often rare and collected pointwise within a larger volume of interest, the mapped structures and properties have to be interpolated or extrapolated throughout this volume. The basic assumption for such interpolation is that properties of the exposed geology are also valid for the hidden geology^1^.

In most underground geo-engineering tasks, the geology is exposed along one or several kilometer-long boreholes. Additional information can be obtained from the interpretation of geophysical imaging^2^, or in the context of high-pressure fluid injections, from seismicity clouds^3^. Both only provide information about large-scale structures. Small-scale information, such as fracture densities and variability of fracture orientations can only be collected from core analysis or geophysical borehole logs, which are time consuming to acquire and require additional economical effort. Nevertheless, the spatial distribution of small-scale fractures and their properties are crucial characteristics of a crystalline rock mass, as these fractures might act as important fluid pathways or could be critically stressed.

Numerical modelling of underground geo-engineering tasks requires - as a fundamental baseline - a detailed geological model that includes geometries and properties of the targeted geological structures. Due to a lack of geological data, most numerical models ignore or strongly simplify the presence of small fractures^4^, which can lead to uncertainties in modelled predictions (e.g., pressure or contaminant propagation in fractured rock). For high-pressure fluid injections for reservoir development e.g., in the context of enhanced geothermal systems (EGS), thermo-hydro-mechanical-seismic (THMS) coupled processes are especially relevant. Data used in EGS research are mostly obtained from laboratory measurements, reservoir-scale stimulations (e.g., Basel, Switzerland^5^ and Soultz-sous-forêt, France^6^) and numerical models^7–9^. In the literature, there is a lack of observations of detailed geological information at the intermediate scale and of the non-linear scaling relationships between rock mechanical parameters. Reducing this data gap might lead to a better understanding of THMS-coupled processes (e.g., onset of hydraulic shearing and fracturing, aseismic vs. seismic slip displacement, pressure diffusion, etc.) and more accurate corresponding numerical models. More comprehensive geological databases for such geo-engineering research might also allow to set up precise (hydraulic) discrete fracture networks (DFNs), to validate the uncertainties in the geological model, and further develop numerical methods for modeling high-pressure fluid injection at high spatial resolution.

In the framework of the decameter-scale in-situ stimulation and circulation (ISC) experiment, a crystalline rock mass was characterized in great detail including geological, hydraulic^10^ and geophysical investigations^11^, as well as in-situ stress measurements^12^. The ISC experiment was conducted at the Grimsel Test Site (GTS), Switzerland, and aimed to fill the research gap for THMS-coupled processes at the intermediate scale. The two endmember methods for permeability creation through hydraulic stimulation (i.e., hydraulic shearing and hydraulic fracturing) were studied during the experiment, as they are key for EGS development^13^. The GTS is operated by Nagra and hosted approximately 480 m below surface in the crystalline rocks of the Central Aar Granite and Grimsel Granodiorite. These rocks are assumed to represent a suitable analogue for the deep crystalline basement, which is the target of several deep geothermal boreholes in Switzerland and elsewhere.

In this contribution, we present a unique geological dataset that has been compiled for this experiment and which was essential for the experimental design and the analysis of observations made during the experiment. We were able to reduce the amount of simplifications for our geological model, due to the extensive geological rock mass characterization from tunnels and fifteen boreholes. We present a 3D visualization of our geological interpretation of the test volume, including the geological architecture (i.e., structures and their properties), which can be expanded towards a discrete fracture network, or implemented in hydrogeological, mechanical, hydro-mechanical or THMS-coupled numerical models. Linked to this publication, we make the geological raw data available to the geological community, as well as our visualization tool for the geological rock mass analog. Thus, the published geological dataset and visualization can be used to study various hydro-mechanical or THMS-coupled processes in a decameter-scale crystalline rock mass independent of a fixed geographic location, thereby advancing research of THMS-coupled processes.

## Methods

In the framework of the ISC experiment an extensive rock mass characterization program was performed to determine geological structures and their thermal-hydro-mechanical-seismic properties:

- *Geology and rock mass structure:* The geology and rock mass structure were characterized based on tunnel wall mapping, core logging, geophysical borehole imaging, i.e. with optical televiewer (OPTV) and acoustic televiewer (ATV) logs, as well as geophysical borehole logging, i.e., seismic velocity, conductivity and resistivity logs.
- *Geophysics:* The geophysical prospecting included seismic tomography between the West and East tunnels, and ground-penetrating radar between boreholes and from tunnel-walls.
- *Hydraulics:* Multi-scale single- and cross-hole hydraulic tests at various borehole intervals revealed hydraulic properties of the rock mass and identified hydraulically important geological structures.
- *In-situ stress measurements:* The in-situ stresses were characterized combining stress-relief and hydraulic methods.
- *Laboratory tests:* From uniaxial and triaxial compression tests combined with active seismic measurements, seismic velocities, dynamic elastic parameters and intact rock permeability were estimated. Additionally, static elastic rock mechanical parameters have been measured.

The ensemble of all these investigations allows the construction of a comprehensive hydro-mechanical geological model of the test volume. Here, we only focus on the baseline geological dataset of the test volume, which includes supplementary basic information from hydraulic and geophysical measurements. Table 1 summarizes the results of all characterization methods and provides information whether the data are published in this contribution or elsewhere.

### Test site and geological background

**Figure 1:**
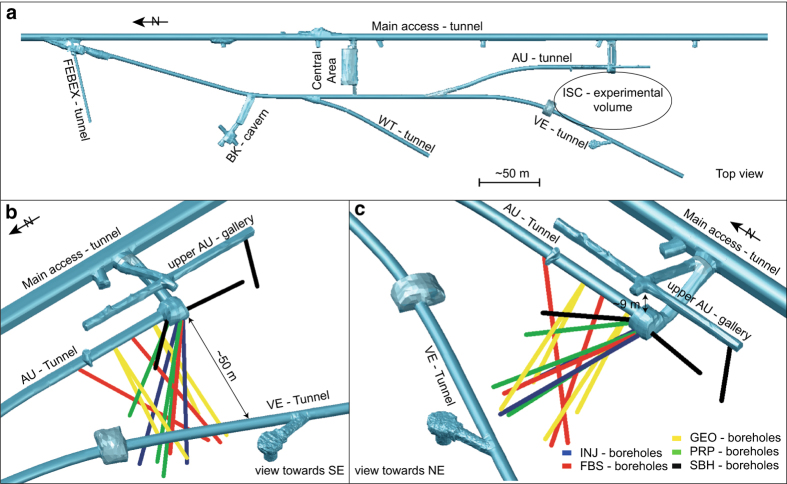
Overview of the GTS and ISC-experimental volume, with boreholes included for the ISC experiment.

The ISC volume can be accessed by three tunnels (Fig. 1) which allow to collect geological information of structures in 3D: The AU-tunnel and the upper AU-gallery in the East and the VE-tunnel in the West. Whereas the AU- and VE-tunnels are TBM tunnels, the upper AU-gallery is a drill-and-blast excavation. A total of 15 boreholes were drilled from AU-cavern (8), AU tunnel (6), and upper AU-gallery (1) into the test volume. The boreholes, with diameters ranging from 86 mm to 146 mm, provide primary point/line information inside the experimental volume. Table 2 summarizes the purposes of the boreholes.

The geology of the Grimsel area has been studied intensely, including detailed investigations of mineralogy, deformation history, and structural geology^14–18^. Our geological model is embedded into these larger-scale geological observations and is based on additional and more detailed rock mass characterizations to meet the requirements for design and interpretation of the in-situ hydraulic stimulation experiment.

The Grimsel area mainly consists of the Variscan intrusive and Post-Variscan metamorphic lithologies of the Aar Massive. The GTS is located at the boundary between the Central Aare Granite (CAGr) and the Grimsel Granodiorite (GrGr)^19^. Both lithologies consist of plutonic rocks of the Early Permian age that intruded the crystalline continental crust 299 ± 2 Ma ago^20^. The rocks of both lithologies show a quartz content between 15–30%^21^ and are close to the mineralogical transition between granodioritic and granitic, with respect to plagioclase content^14,15^. The main difference between the two lithologies is the amount of sheet silicate minerals (such as biotite and white mica)^17^. Zones of weakness from higher sheet silicate contents emerged during the latest phase of differentiation of the plutonic bodies. Along these zones, aplitic dykes and lamprophyres intruded^22^. Pre-Alpine deformation structures are not identified in either lithology.

During Alpine deformation, the rocks were metamorphosed under upper greenschist facies conditions with peak-temperatures of 450 ± 30 °C and peak-pressure of 6 ± 1 kBar^23,24^. Note, that newer studies have indicated that peak pressures were probably around 4.4 kBar^25^. Alpine metamorphism transformed the lamprophyres into meta-basic dykes with enriched biotite content^17^. Additionally, deformation caused a reorientation of sheet silicates forming a pervasive foliation in both lithologies, with a prevailing strike of N50°E and a dip of 80° towards SE^26^.

The CAGr and GrGr were also deformed in a purely ductile, brittle-ductile and purely brittle manner during Alpine orogeny. Three different shear zones with respect to orientation and relative age have been distinguished^14^: 1) The oldest shear zones strike on average N52°E and dip with 77° towards the southeast. These are purely ductile (with very few parallel brittle fractures). 2) The second shear zone set is slightly discordant to the oldest one, striking N67°E with a dip of 75° towards the southeast, and is ductile, too. This orientation also corresponds to the alpine foliation. 3) The youngest shear zones have a strike of N93°E and a dip of 65° towards the south and are brittle-ductile. Previous studies classified those shear zone directions as S1, S2, and S3, respectively^14^. Note, that S1 and S2 cannot be distinguished in the field^14^. The brittle faulting happened afterwards (less than 10 Ma ago) and formed cataclasites, breccias, and clay fault gouge^18^.

### Geological characterization

In a first phase of the fieldwork, all three tunnels were mapped with the support of geodetic measurements, focusing on large structures (i.e. shear zones, dykes, persistent fractures). Three coordinate points were measured along fractures, and six coordinate points along shear zones and dykes, with three coordinates on each boundary. Planes fitted through three related coordinates provided a measure of dip-direction and dip of the structures. A total of five major shear zones was mapped along the AU- and VE-tunnels, which can be divided into two groups with respect to orientation and mineralogy/structure. Following previous studies^14^, the shear zones were classified as *S1* (SE-dipping ductile) and *S3* (S-dipping brittle-ductile, i.e., ductile shear-zone with late phase brittle deformation) shear zones.

**Figure 2:**
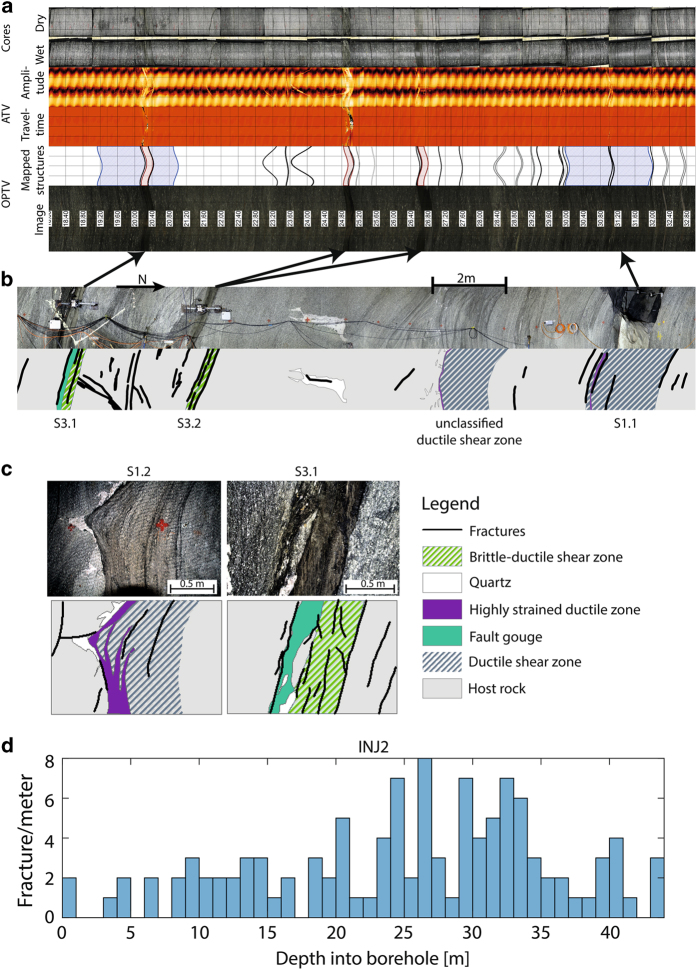
Example results from geological mapping of borehole INJ2 and the AU tunnel wall. (**a**) From top to bottom: core optical images (i.e., dry and wet conditions), ATV logs (i.e., amplitude and travel time), OPTV logs (i.e., mapped structures and image), arrows connecting shear zones mapped along tunnel walls with locations in boreholes. (**b**) Panorama image of AU tunnel wall section along with schematic map. (**c**) Schematic geological maps of both shear zone types are shown in higher resolution below. (**d**) Measured fracture density from core and borehole logging.

In addition to the geodetic mapping, the architecture of the shear zones was mapped in detail based on panoramic photographs and detailed images of relevant structures (Fig. 2). These visualizations illustrate the difference in structure between the two shear zone types. They also highlight that each shear zone is a 3D feature that varies in thickness throughout the entire volume. The average thickness varies between 173 mm and 1670 mm for *S1,* and 38 mm and 312 mm for *S3*. The *S1* shear zone direction indicates NW-directed thrusting with localized high strain zones isolating lenses of reduced deformation^17^. Due to this effect, many authors^27–29^ have described this type of shear zone as anastomosing. Additionally, it has to be considered, that brittle fractures branch from these ductile shear zones, which are partly filled with quartz. The *S3* shear zone direction contains evidence for dextral strike-slip movement^14,17^. Whenever the orientation of the meta-basic dykes aligns with the *S3*-direction, these brittle ductile shear zones localize within those dykes. Brittle fractures, as well as milky quartz veins, started forming during this phase of *S3*-oriented shearing^26^. Those fractures are often covered with biotite, indicating that the brittle deformation took place under low greenschist facies conditions, where biotite could grow in a chemically stable manner^26^. Note that we considered thin mechanical discontinuities as fractures, regardless if they showed secondary mineralization or not.

The *S1* and *S3* shear zones are persistent structures that can be traced over a distance of around 500 m to the surface^19^. Both shear zones have a core (i.e., zone of highest deformation) and an asymmetric habitus. The core of the *S1* shear zones is at their southern boundary (Fig. 2). In the *S3* shear zones, fault gouge is not well localized throughout the shear zones, and the most persistent fractures (i.e., which are also hydraulically important) are located at the boundary between shear zone and host rock. The persistence of the structures makes the test volume a suitable analog rock mass for deep geothermal exploitation, since it is assumed that discrete large-scale structures (i.e., shear zones or fractures) are crucial for EGS development^6^.

The extracted cores from the boreholes were photographed in dry and wet conditions. The core diameters can be found in Table 2. The pictures were transformed into ortho-images and referenced with respect to the in-situ depth along the borehole axis of the extracted core. In a final processing step the pictures were stitched, to produce complete core logs for each borehole. The main focus of these logs is counting fractures and localization of shear zones and dykes. For the fractures, we could clearly distinguish between drilling induced (i.e., normal to borehole axis, no mineral cover, circular scratches on surfaces) and natural fractures. Combining the core logs with optical televiewer (OPTV) and acoustic televiewer (ATV) logs yielded a detailed fracture density dataset for all boreholes. The natural fracture density in the host rock varies between 0 and 3 fractures/meter and increases towards >20 fractures/meter between the *S3* shear zones. We refer to this area between the *S3* shear zones as the “highly fractured zone”. Additionally, OPTV and ATV logs were used to gather information about true orientations of fractures, shear zones and foliation in the test volume. According to our data, the *S3* shear zones consistently dip towards south. OPTV logs revealed a change in orientation of *S1* shear zones inside the volume. This re-orientation of the *S1* shear zones fits the earlier observations^17^ that the *S1* shear zone experienced dextral dislocation along the *S3* shear zone.

### Borehole geophysical methods

In addition to the aforementioned borehole imaging logs, several geophysical borehole logs were obtained to characterize the rock mass (see Table 3). The resistivity logs (GuardLog) were used to roughly localize zones of increased fragmentation, as well as to characterize the shear zones. Changes in resistivity along the borehole are interpreted as fluid filled fractures or shear zones. The resistivity values range between 2000 and 3000 Ωm for the *S3* shear zones, between 5000 and 6500 Ωm for the *S1* shear zones and exceed 10000 Ωm for the intact host rock. The values indicate that fracturing and micro-fracturing is more intense in the S3 shear zones compared to the S1 shear zones. Note, that the resistivity values for the intact host rock were clipped, since they exceeded the maximum detection level of the used probe.

The seismic velocity logs were acquired using a novel borehole tool consisting of one piezo-electric source at the tip and three piezo electric receivers at distances of 0.5, 1.0 and 1.5 m from the source. The tool was moved along the boreholes and measurements were taken every 0.25 m. At each survey location, source and receivers were pneumatically clamped to the borehole wall to ensure coupling so that the probe does not rely on borehole fluid for successful signal transmission. P- and S-wave arrivals could be reliably identified and were manually picked for all data. These P- and S-wave arrival times were used in 1-D travel time inversions that result in P- and S-velocity estimates along each borehole. We found that the travel time inversions give comparable results to a semblance analysis but are more robust in the area of the shear zones, where the waveforms are variable between traces due to the local heterogeneity of the rock.

Flowing fluid electrical conductivity (FFEC) logs were conducted in the framework of hydraulic cross hole testing. These logs were used to trace hydraulically activate fractures by monitoring changes in fluid conductivity due to fresh water inflow into flushed monitoring boreholes. Prior to testing, the electrical conductivity of the monitoring borehole fluid was increased by flushing with salt water.

### Rock mechanical parameters

Along with the geological characterization, we investigated the rock mechanical parameters of the rock mass, including a literature review, and static and dynamic laboratory measurements. The elastic parameters were determined for the host rock, as well as for the meta-basic dykes and fractures.

Keusen *et al.*^14^, collected statically measured elastic rock properties in the framework of their geological mapping and characterization of the Grimsel Test Site (see Table 4). The measurements were conducted on intact drill cores under uniaxial conditions. The properties of the actual rock mass vary due to their dependence on rock type.

Vogler *et al.*^30–32^, measured static elastic parameters of the granitic matrix and fractures in uniaxial configuration. Depending on the tested specimen size they measured tensile strength ranging from 6 MPa (300 mm diameter specimen) to 11 MPa (54 mm diameter specimen) using Brazilian tests. Additionally, they measured static Young’s-Moduli ranging between 10 GPa and 12 GPa for fractured specimens.

In addition to static measurements, dynamic measurements of the rock mechanical properties were conducted in-situ ^33^ and in the laboratory^21^ (see Table 5). In combination with an assumed density of 2700 kg/m^3^ the in-situ dynamic elastic parameters can be calculated from the seismic streamer logs. The laboratory measurements were conducted under 30 MPa confining pressure. Note that the in-situ stress level is lower with σ_3_ ranging from less than 2.8 MPa (perturbed stress field close to shear zones) to 9.7 MPa (unperturbed ‘far-field’ stress field)^34^, and the mean stress (σ_mean_) varying between 9.2 MPa and.10.3 MPa.

### Geological interpolation and visualization

Based on the compiled geological dataset, we built a Matlab based visualization tool which is published with the dataset via ETH research collection (Data Citation 1). Within this tool, we plot all mapped fractures and shear zones at the boreholes with true dip-direction and dip. Additionally, we plotted the interpolated shear zones in the volume.

To interpolate the five major shear zones throughout the entire test volume we conducted three processing steps. Since each of these steps represents an interpretation of data, we provide the raw data and interim results in the database. Note that our interpolation method was based on fitting planes/surfaces through the midpoints of each shear zone. Thus, our interpolation and the resulting visualization do not include information about the thickness and architecture of the shear zones. This information can be found separately for each tunnel and borehole in the published database.

As a first step for the interpolation, we arranged the mapped shear zone coordinates obtained from tunnels and boreholes into specific sets. Each set represents a specific shear zone (i.e., S1.1, S1.2, S1.3, S3.1 & S3.2). To arrange the data into sets, we considered true dip directions and dip angles.

The second processing step was a linear connection between groups of three coordinates within each set (i.e., triangulation). For this interpolation we mapped linear patches for each group of three coordinates. Since the edges of the patches followed the closest connection line between two coordinates, no true local orientation measurements were considered in this step.

As last processing step, we integrated true local shear zone orientations into the interpolation and created additional coordinates for each shear zone between the boreholes. To do so, we assumed that the shear zone orientations were constant within a radius of 5 m around the tunnels (i.e., measured from the tunnel center) and within a radius of 0.5 m around the boreholes. Following this assumption, we calculated additional shear zone coordinates along discs of the given radii and corresponding orientation around each tunnel/borehole. Then all boreholes and tunnels were connected using a third order polynomial function which additionally was fitted to the discs of constant orientations. We calculated 20 additional coordinates along each third order polynomial function. Subsequently, surfaces were fitted through all corresponding coordinates for each shear zone, including true data points (i.e., mapped coordinates) and interpolated coordinates.

### Visualization tool

The visualization tool shows all data in a coordinate system, which has its origin with respect to the Swiss coordinate system origin at

X (Easting) = 667400

Y (Northing) = 158800

Z (Elevation) = 1700.

The tool shows the different steps of data interpretation. It shows the raw data, the linear interpolated shear zones, and the 3^rd^ order polynomial interpolation. Additionally, the fractures are shown in 3D at the boreholes locations and fracture density is visualized. Within the tool, all raw data (e.g., borehole paths and geological data) are linked from .txt-files. Thus, a change in borehole trajectory or change in fracture location along a borehole will be automatically updated in the figure.

### Code availability

**Figure 3:**
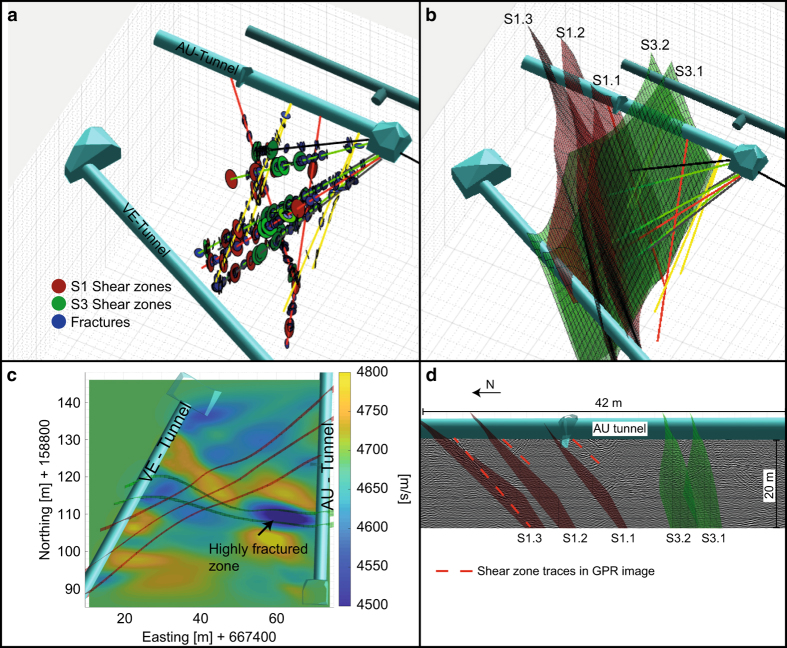
Visualization of the geological model including geological raw data, interpolated shear zones and geophysical data. (**a**) Mapped geological data along boreholes; (**b**) Final interpretation of the geological model; (**c**) Seismic tomography between AU- and VE Tunnel with indicated S3-shear zones and highly fractured zones; (**d**) GPR measurements from AU-tunnel with indicated S1-shear zones.

All data were processed with non-custom-codes. The only custom code that was used in this study was for the 3D visualization of the geological data. This code was built in Mathworks Matlab R2018a and can be download from ETH Zurich Research Collection (Data Citation 1). To run the code, one just needs to open the file “Geological_model_visualization.m”. Within this code several sub-functions that are stored in the functions-folder are implemented. Thus, everything can be modified by the user. If the code is run without any modification it produces, in addition to others, the pictures in Fig. 3a and b.

## Data Records

The dataset is published via ETH Zurich Research Collection (Data Citation 1). The dataset is built as a folder structure in which we separated basic input data (i.e., tunnel and borehole coordinates) and geological observations (i.e., core- and borehole logs, fracture densities), from interpreted data (i.e., shear zone interpolations). Table 1 summarizes the data and the file-formats that are provided in the dataset. It also lists additional data and corresponding publications. In addition, we published a separate folder, that contains the Matlab code, functions and required input data to build our 3D visualization.

All data are stored in .txt and .png-files. The only exception are the borehole logs, which are stored in .wcl files. To open these files the commercial software *ALT WellCAD*, or its freeware version called *ALT WellCAD reader* can be used. The WellCAD reader can be downloaded from https://www.alt.lu/downloads.htm and an executable of the current version (January 2018) is available within the borehole logs folder in the database. Note that the .wcl-files were uploaded separately into the repository, due to their size.

Within all main folders we stored readme-files, that explain the structure of the data contained in the folder.

## Technical Validation

To test the validity of the geological model, it was compared with independent geophysical and hydrogeological observations. Both methods were used to confirm the interpolation of the large-scale features (i.e., shear zones), geological observations such as degree of fragmentation as well as rock physical properties.

### Geophysical methods

Seismic tunnel-tunnel measurements were performed between the AU- and VE-tunnels for the purpose of travel time tomography. 123 hammer shots (spacing: 0.5 m) at the western sidewall of AU-tunnel were used as seismic sources and recorded with 120 geophones (spacing: 0.5 m) on the eastern sidewall of VE-tunnel. More than 10000 travel times were used in an anisotropic travel time inversion assuming the axis of symmetry parallel to the foliation of the rock. The velocity along the symmetry axis is shown in Fig. 3c. The strongest feature in the seismic p-wave model is a low velocity zone located in the eastern part of the zone between the S3 shear zones. This is in agreement with borehole observations, which characterize this zone as strongly fragmented (i.e., highest frac/meter ratio). The S3 shear zone is known to have a brittle component and is associated with fractures (see above). Therefore, it can be traced through the test volume using the seismic tomography. The traces are in good alignment with the geological interpolation (Fig. 3c). It is noteworthy that the seismic velocity – and thus possibly the degree of fracturing – appears to be more intense towards the east of the intersection between S1 and the S3 structures. The S1 shear zones do not show a p-wave velocity variation evident in the seismic tomogram possibly because they are purely ductile.

Additionally, ground penetrating radar reflection data were acquired from the AU-tunnel looking westwards using shielded 160 MHz antennas. Figure 3d shows the migrated data following standard processing steps^33^. The S1 shear zones (i.e., S1.1, S1.2, and S1.3) can be clearly identified and be traced from the AU-tunnel walls into the volume. The most obvious traces correlate with the southern boundaries of the interpolated shear zones. From the geological mapping, it is known that the southern boundaries of the shear zones contain highly strained zones (Fig. 2). Thus, the GPR images supported the interpolation of the geological interpolation of the S1 shear zones, as well as the observation of highly strained southern boundaries. Since the S3 shear zones are perpendicular to the AU-tunnel, and thus to the profile along which the GPR measurements were conducted, they are not visible in the reflection image.

### Hydrogeological properties

Hydraulic borehole screening was conducted in both INJ-boreholes, as well as in one PRP, one FBS and one SBH borehole. Systematic pulse injection tests in 2 m packer-intervals were performed in intervals with high fracture density. This screening allowed us to identify permeable fractures and shear zones for further hydraulic characterization with constant-rate injection tests under tomographic conditions. Due to the given packer-interval size (2 m) and the given fracture density (minimum 0−3 fractures per meter), it was not possible to test single fractures. This screening confirmed, that discrete discontinuities are the most important factor for pressure diffusion in the studied rock mass.

The crystalline rock at GTS is saturated and most of the natural permeability occurs in shear zone parallel (*S3* orientation rather than *S1* orientation) fractures, and especially along meta-basic dyke/host rock contacts. The observed in-situ pressure in the major shear zones in the ISC experimental rock volume is around 0.2–0.3 MPa due to long-term drainage of the near-by tunnels. The average interval transmissivity of the intact rock is in the range of 10^−14^ – 10^−13^ m^2^/s, whereas the transmissivity of shear zones ranges from 10^−12^–10^−6^ m^2^/s ^14,35^. The highest transmissivities were measured in several packer-intervals that were either along the boundary between shear zone and host rock, or close to the shear zones in the host rock. This might be an effect of the increase in fracture density towards the shear zones, and the high fracture concentration between the two *S3* shear zones which is also consistent with the drop in electric resistivity within these zones. Five-packer systems were installed in the two INJ-boreholes to conduct cross-hole pressure tomography. The obtained injectivities vary between 0.003 ml/min/kPa – 0.84 ml/min/kPa with the exception of 48.3 ml/min/kPa at the *S3* shear zone in the INJ2 borehole^36^. The specific storage varies in a range of 10^−9^–10^−6^ m^−1^ ^37^. A detailed analysis of all conducted pulse tests and further hydraulic characterization tests is work in progress and will be published separately.

Prior to detailed cross-hole hydraulic tests, fluid electrical conductivity (FFEC) logging and heat tracer tests were implemented between two injection boreholes. The initial conditions of the monitoring boreholes were perturbed by replacing borehole water with higher electrical conductivity fluid (FFEC logging) or warming up the water in the monitoring borehole (thermal perturbation test). The intrusion of in-situ water through the natural fractures is then measured via electrical conductivity borehole loggers and/or distributed temperature fiber optics. The following information was obtained under two test conditions:

- Natural condition, where the other borehole is packed off and the location of water intrusion in monitoring borehole reflects the depth of producing fractures.
- Forced condition, where the water was injected into the other INJ borehole with a constant pressure (<5 bars) and the intrusion of in-situ water in monitoring borehole was monitored. In this case, the depth of the intrusions reflects the location of most conductive fractures between these two boreholes.

Since the used packer interval ranged from 1 to 6 m intervals in various boreholes, it was not trivial to test the connectivity of single structures, so instead the hydraulic properties of shear zones and their adjacent fractures were analyzed and constrained. These data validated that the *S3* shear zones with the adjacent parallel fractures - especially the highly fractured zone in between the *S3* shear zones - are hydraulically important structures that are persistent throughout the entire volume. The hydraulic data do not validate the overall shear zone interpolations but show that the five interpolated shear zones and the nearby persistent fractures are the most important hydraulic structures within the volume. Nevertheless, the true fluid pathways within the test volume (i.e., along the true fracture network) need to be visualized within a hydraulic discrete fracture network (i.e., HydroDFN), which is recent work in progress and will be published separately.

The comparison between the geological model and independent obtained geophysical and hydrogeological data confirmed that the interpolation of the shear zones is valid. Geological features, such as fracture density, correlate well with geophysical observations. Additionally, it was shown that the geological persistent structures and highly fractured zone around the S3 shear zones are the hydraulically important structures, while the ductile S2 shear zones are much less hydraulically conductive and connected. We argue that the characterized rock mass serves as a valid analog volume for a crystalline basement rock mass.

## Additional information

**How to cite this article**: Krietsch, H. *et al*. Comprehensive geological dataset describing a crystalline rock mass for hydraulic stimulation experiments. *Sci. Data*. 5:180269 doi: 10.1038/sdata.2018.269 (2018).

**Publisher’s note**: Springer Nature remains neutral with regard to jurisdictional claims in published maps and institutional affiliations.

## Supplementary Material



## Figures and Tables

**Table 1:** Overview of characterization methods and datasets.

Category	Characterization	Detail	Stored as/published in
Geology	Tunnel mapping	Geodetic mapping	.txt-files
		Panoramic pictures with mapped shear zones and fractures	.jpg-files
		Detailed pictures of exemplary shear zones to display different characteristics	.jpg-files
	Core logging	Pictures in wet conditions	.jpg-files with depth reference and included in a .wcl-file
		Pictures in dry conditions	.jpg-files with depth reference and included in a .wcl-file
		Fracture density (combined with OPTV data)	.txt-file and .png-files with histograms
	Geophysical borehole logging	OPTV logs	.wcl-file and .txt-file with exported mapped geological structures including location and true orientation
		ATV logs	.wcl-file
		Resistivity logs	.wcl-file
		Seismic velocity logs	.wcl-file
Hydrogeology	Borehole scale	Single hole packer tests (Pulse, constant rate and head)	*Jalali et al. a*^35^*; Brixel et al.*^38^
		Periodic injection test	*Brixel et al.*^39^
	Cross-hole scale	Flowing fluid electrical conductivity (FFEC)	*Jalali et al.*^36^
		Thermal fluid logging	*Jalali et al.*^36^
		Periodic interference injection test	*Brixel et al.*^38^
		Crosshole packer tests (Pulse, constant rate and head)	*Brixel et al.*^38^
		Hydraulic tomography	*Jalali et al.*^36^
	Reservoir scale	Long term injection test	*Brixel et al.*^40^
		Conservative (dye, salt and DNA-nano) tracer test	*Kittilä et al.*^41^*; Kittilä et al.*^42^
		Thermal tracer test	*Brixel et al.*^40^
		Salt/ethanol tracer test with GPR	*Giertzuch et al.*^43^
Geophysics	Seismic	Tomography between AU and VE tunnel	.png-file
	GPR	Measurements from tunnel walls	.png-file *Doetsch et al.*^11^
		Measurements between boreholes	*Doetsch et al.*^11^
In-situ stress measurements	Characterization of ‘far-field’ and perturbed stress field	Stress-relief (i.e., overcoring) and hydraulic (i.e., hydraulic fracturing) methods	*Krietsch et al.*^12,34^
Laboratory	Petrophysical properties (dynamic)	Characterizing the anisotropic elastic and fluid flow properties on shear zone and host granodiorite samples.	*Wenning et al.*^21^
	Static elastic parameters	Young’s modulus and tensile strength of intact rock	*Vogler et al.*^30^ *Vogler et al.*^31^
		Stiffness of fractures	*Vogler et al.*^32^
	Hydraulic parameters	Permeability of matrix and fractures	*Vogler et al.*^32^ *Vogler et al.*^30^
The presented geological datasets of this contribution are listed with the file types as which they are stored.			
References are provided for data and results that are not included in this dataset.			

**Table 2:** Overview of boreholes within the ISC test volume.

Borehole Type	Borehole Diameter [mm]	Core Diameter [mm]	Borehole Lengths [m]	Main Purpose	Characterization Methods
INJ (2x)	146	120	44.66 m–44.80 m	Injection & pressure monitoring	Core-logging, borehole imaging, geophysical borehole logging, hydraulic tests
FBS (3x)	101	85	44.00 m–47.58 m	Strain monitoring	Core-logging, borehole imaging, hydraulic tests
GEO (4x)	86	75	30.10 m–40.09 m	Active/passive seismic monitoring	Core-logging, borehole imaging, geophysical borehole logging
PRP (3x)	131	110	32.33 m–47.91 m	Pore-water pressure and strain monitoring	Core-logging, borehole imaging, hydraulic tests
SBH (3x)	101	85	18.20 m–23.90 m	Stress measurement	Core-logging, borehole imaging, stress measurement campaign, hydraulic tests

**Table 3:** Overview of conducted geophysical borehole logs.

Log-name	Measured Boreholes
Optical Televiewer (OPTV)	All boreholes
Acoustic Televiewer (ATV)	INJ- and GEO-group
Resistivity log (GuardLog)	INJ-group
Seismic velocity logs	INJ- and GEO-group
Flowing fluid electrical conductivity (FFEC)	INJ-group

**Table 4:** Static rock mechanical parameters.

	Central Aar Granite	Grimsel - Granodiorite	Meta-basic dykes
Volumetric weight [kg/m^3^]	2660 ± 23.8	2706 ± 13.6	2909 ± 31.0
Porosity [Vol. %]	0.4 – 1.0		
E-Modulus [GPa]	53.3 ± 11.0	47.3 ± 15.4	42.4 ± 8.5
Poisson’s Ratio	0.37 ± 0.12	0.33 ± 0.15	0.33 ± 0.17
Tensile strength [MPa]	9.06 ± 1.48	9.54 ± 2.17	12.55 ± 3.59
Uniaxial compressive strength [MPa]	169.1 ± 37.1	116.9 ± 47.9	127.0 ± 31.8
Friction angle [°]	33	30 ± 2	32.5 ± 3.5
Adapted from Keusen *et al.*^14^.			

**Table 5:** Results from geophysical borehole logs and laboratory tests listing P-wave velocity (V_p_), S-wave velocity (V_s_), Dynamic Poisson ratio (*υd)*, Dynamic Young’s Modulus (*Ed*), and Dynamic Bulk Modulus (*Kd*).

	V_p_ [m/s]	V_s_ [m/s]	*υ*	*Ed* [GPa]	*Kd* [GPa]
Grimsel –Granodiorite *In-situ*^a^	5120–5171	2678–2725	0.31–0.33	47–51	42–46
S1-shear zone *In-situ*^a^	4953–4921	2430–2500	0.33–0.34	40–43	42–44
S3-shear zone *In-situ*^a^	4568–4670	2530–2580	0.27–0.3	42–45	32–40
Grimsel – Granodiorite *Laboratory*^b^	5300–5340	3320–3370	0.16–0.19	65–81	31–41
^a^After Doetsch *et al.*^33^,					
^b^After Wenning *et al.*^21^.					

## References

[d1] ETH Zurich Krietsch H. *et al.* 2018 https://doi.org/10.3929/ethz-b-000243199

